# 

**Published:** 1904-05-07

**Authors:** 


### NOTICE TO CORRESPONDENTS.

All MSS., Letters, Books for Review, and other matters intended for the Editor should be addressed The Editor, "The Hospital," The Hospital Buildings, 28 & 29 Southampton Street, Strand, W.C.

The Editor cannot undertake to return rejected MS., even when accompanied by stamped directed envelope.

**Notice to Advertisers and Subscribers.**

All Advertisements, Orders for Copies of The Hospital, and Business Communications should be addressed to **THE MANAGER (Not to the Editor),** The Hospital, 28 & 29 Southampton Street, Strand, London, W.C.

The Cost of Subscription, post free, is as follows:—Per week, 3½d.; per quarter, 3s. 3d.; per half-year, 6s. 6d.; per annum, 13s. Foreign and Colonial:—Per annum, 19s. 6d., payable, in all cases, in advance.

**NOTICE.—Vol. XXXV. of "The Hospital" (October 1903 to March 1904), in handsome cloth binding, is now on sale at the office of this Journal, price 10s. 6d. Cases for binding the Numbers contained in this Volume 2s. Index to Vol. XXXV., 3½d., post free.**

**The Monthly part for May is now ready. Price 1s., by post, 1s. 3d.**

### BOOKS RECEIVED.

John Bale, Sons and Danielsson, Ltd.
"Handbook of Gynæcological Pathology." By C. H. Roberts

J. Hedderwick and Sons.
"Knights Hospitallers in Scotland." By G. T. Beatson.

W. B. Saunders and Co.
"Obstetrics for Nurses." By J. B. de Lee, M.D.

Scientific Press, Ltd.
"A Complete Handbook of Midwifery." By Dr. J. K. Watson.

### The Student of Medicine.

### APPOINTMENTS.

**G. Stewart Abram**, B.A., M.B., B.C.Cantab., M.R.C.S.Eng., L.R.C.P.Lond., Junior Assistant Physician to the Royal Berkshire Hospital. **W. F. C. Bennett**, L.R.C.P.&S.Edin., District Medical Officer of the Sheffield Union, **James Burnet**, M.A., M.B., M.R.C.P.Edin., Registrar to the Royal Hospital for Si[illegible] Children, Edinburgh. **G. M. Dawkin**, M.R.C.S., L.R.C.P.Lon[illegible] District Medical Officer of the Pontypridd Union. **W. S. Eato[illegible]** M.B., Ch.B.Edin., District Medical Officer of the Whiteha[illegible] Union. **D. G. Hall**, M.D., B.C.Cantab., House Physician to [illegible] Sussex County Hospital, Brighton. **J. D. Hendry**, [illegible] C.M.Aberd., District Medical Officer of the Poplar Union. [illegible] **Hutchinson**, M.R.C.S., L.R.C.P., Medical Officer of the Canno[illegible] Union Workhouse. **T. D. Kirk**, M.D., Ch.B., R.U.I., Medi[illegible] Officer of the Workhouse and Public Vaccinator for the Berri[illegible] District of the Forden Union. **Edward E. Norton**, M.R.C.[illegible] L.R.C.P., D.P.H., Medical Superintendent of the Brentford Un[illegible] Infirmary. **W. H. O'Meara**, M.R.C.P., L.R.C.S.Irel., Medi[illegible] Referee under the Workmen's Compensation Act for Cou[illegible] Carlow. **T. C. Orford**, M.R.C.S., L.R.C.P.Lond., District Medi[illegible] Officer of Grenada, West Indies. **S. H. Smith**, M.B., M.S.Edi[illegible] Medical Officer of the York Union Workhouse. **A. R. Spence[illegible]** M.D.Lond., M.R.C.S., L.R.C.P., Resident Medical Officer to t[illegible] Hospital for Epilepsy and Paralysis, Maida Vale. **J. R. H[illegible] Walker**, M.D.Edin., Medical Officer to the Post Office at Have[illegible] including Thurlow. **B. J. E. Wright**, L.R.C.P.&S.Edi[illegible] L.F.P.S.Glasg., District Medical Officer of the Ellesmere Union.

**"The Hospital" Institutional Library.**

"Hospitals and Asylums of the World." 4 Vols., with a Po[illegible] folio of Plans, complete £12 12s.

"Burdett's Hospitals and Charities, 1903." 5s. net.

"Burdett's Official Nursing Directory, 1903." 8s. net.

"Cottage Hospitals, General, Fever, and Convalescent." 10s. [illegible]

"Uniform System of Accounts for Hospitals and Public Instit[illegible] tions." New Edition. 4s. net.

"Hospital Expenditure: The Commissariat." 2s. 6d.

"A Legal Handbook for the Use of Hospital Authoriti[illegible] 2s. 6d.

"The Cottage Hospital Case Book or Register of Patients." 1[illegible] and 12s. 6d.

All these are published by the Scientific Press, Ltd., and m[illegible] be obtained through any bookseller, or direct from the publishe[illegible] 28 and 29 Southampton Street, Strand, London, W.C.

JUST PUBLISHED.

### A COMPLETE HANDBOOK OF MIDWIFERY.

By J. K. WATSON, M.D., M.B., C.M.

Author of "A Handbook for Nurses," "Examination of the Urine," &c., &c.

This handbook has been designed to meet the requirements of the Central Midwives Board, and is an up-to-date text-book of **all** that the midwife requires to know of the subject treated. The work is produced with the object of rendering it unnecessary for midwives, during their preparation for examination, to consult the larger and more expensive works on the subject written specially for medical men.

**The illustrations are very complete, numbering some 143 separate diagrams, most of which have been specially prepared for this book.**

Crown 8vo., about 350 pp., bound in cloth boards, **6/-** net, **6/4** post free.

NOW READY.

### THE MIDWIVES' POCKET BOOK.

By HONNOR MORTEN.

With Appendix containing particulars and rules of the Central Midwives Board.

Bound in cloth, 1/- post free.

| | | |
|---|---|---|
| 2/- Post Free. | **PRACTICAL GUIDE TO SURGICAL BANDAGING AND DRESSINGS.** By Wm. Johnson Smith, F.R.C.S. Demy 16mo. profusely illustrated (suitable for Apron pocket), cloth - - - - - - - - | 2/- Post Free. |
| 1/- net, 1/1 Post Free. | **THE EXAMINATION OF THE URINE.** By J. K. Watson, M.D., M.B., C.M., Author of "A Handbook for Nurses." Demy 16mo. cloth boards - - - - - - | 1/- net, 1/1 Post Free. |

*Of all Booksellers, or of*

THE SCIENTIFIC PRESS, LTD., 28 & 29 Southampton Street, Strand, London, W.C.

*Complete Catalogue of Nursing Handbooks post free on application.*

